# Impact of process temperature and organic loading rate on cellulolytic / hydrolytic biofilm microbiomes during biomethanation of ryegrass silage revealed by genome-centered metagenomics and metatranscriptomics

**DOI:** 10.1186/s40793-020-00354-x

**Published:** 2020-03-02

**Authors:** Irena Maus, Michael Klocke, Jaqueline Derenkó, Yvonne Stolze, Michael Beckstette, Carsten Jost, Daniel Wibberg, Jochen Blom, Christian Henke, Katharina Willenbücher, Madis Rumming, Antje Rademacher, Alfred Pühler, Alexander Sczyrba, Andreas Schlüter

**Affiliations:** 1 Bielefeld University, Center for Biotechnology (CeBiTec), Genome Research of Industrial Microorganisms, Universitätsstr. 27, 33615 Bielefeld, Germany; 2 Department Bioengineering, Leibniz Institute for Agricultural Engineering and Bioeconomy (ATB), Max-Eyth-Allee 100, 14469 Potsdam, Germany; 3 Helmholtz Centre for Infection Research, Microbial Infection Biology / Experimental Immunology, Inhoffenstrasse 7, 38124 Braunschweig, Germany; 4 Department Bioinformatics and Systems Biology, Justus-Liebig University Gießen, Heinrich-Buff-Ring 58, 35392 Giessen, Germany; 5 Faculty of Technology, Bielefeld University, Universitätsstr. 25, 33615 Bielefeld, Germany

**Keywords:** Metagenome assembled genomes, Integrated -omics, Polyomics, Anaerobic digestion, Biogas, Bioconversion, Microbial community structure, Methane, Metabolic activity

## Abstract

**Background:**

Anaerobic digestion (AD) of protein-rich grass silage was performed in experimental two-stage two-phase biogas reactor systems at low *vs*. increased organic loading rates (OLRs) under mesophilic (37 °C) and thermophilic (55 °C) temperatures. To follow the adaptive response of the biomass-attached cellulolytic/hydrolytic biofilms at increasing ammonium/ammonia contents, genome-centered metagenomics and transcriptional profiling based on metagenome assembled genomes (MAGs) were conducted.

**Results:**

In total, 78 bacterial and archaeal MAGs representing the most abundant members of the communities, and featuring defined quality criteria were selected and characterized in detail. Determination of MAG abundances under the tested conditions by mapping of the obtained metagenome sequence reads to the MAGs revealed that MAG abundance profiles were mainly shaped by the temperature but also by the OLR. However, the OLR effect was more pronounced for the mesophilic systems as compared to the thermophilic ones. In contrast, metatranscriptome mapping to MAGs subsequently normalized to MAG abundances showed that under thermophilic conditions, MAGs respond to increased OLRs by shifting their transcriptional activities mainly without adjusting their proliferation rates. This is a clear difference compared to the behavior of the microbiome under mesophilic conditions. Here, the response to increased OLRs involved adjusting of proliferation rates and corresponding transcriptional activities. The analysis led to the identification of MAGs positively responding to increased OLRs. The most outstanding MAGs in this regard, obviously well adapted to higher OLRs and/or associated conditions, were assigned to the order *Clostridiale*s (*Acetivibrio* sp.) for the mesophilic biofilm and the orders *Bacteroidales* (*Prevotella* sp. and an unknown species), *Lachnospirales* (*Herbinix* sp. and *Kineothrix* sp.) and *Clostridiales* (*Clostridium* sp.) for the thermophilic biofilm. Genome-based metabolic reconstruction and transcriptional profiling revealed that positively responding MAGs mainly are involved in hydrolysis of grass silage, acidogenesis and / or acetogenesis.

**Conclusions:**

An integrated -omics approach enabled the identification of new AD biofilm keystone species featuring outstanding performance under stress conditions such as increased OLRs. Genome-based knowledge on the metabolic potential and transcriptional activity of responsive microbiome members will contribute to the development of improved microbiological AD management strategies for biomethanation of renewable biomass.

## Background

Important part of bioeconomical strategies for sustainable and carbon dioxide (CO_2_) - neutral energy production is the anaerobic digestion (AD) and biomethanation of renewable raw materials. Beside manure and slurries from agricultural husbandry, also agriculturally produced biomass such as maize silage (‘energy crops’) or material from landscape management were utilized [1, 2].

Crop biomass is rich in long-chained carbohydrates such as cellulose, hemicellulose, and xylan and additionally contains considerable amounts of proteins depending on the particular crop. To digest crop biomass as sole substrate and at high organic loading rates (OLRs), specially designed biogas reactors are advantageous such as staged degradation step (‘phase’) separated reactor systems consisting of a cellulolysis / hydrolysis fermenter and a downstream methanogenesis reactor. Compared to single phase reactors, these two-stage two-phase reactors possess several advantages, for example, the more stable operation (under particular process conditions) combined with higher bioenergy yields [2–6].

The degradation of high-molecular compounds requires the direct access of microbial specialists. Accordingly, crop biomass is colonized by a cellulolytic biofilm which composition varies with the abiotic environmental conditions, as example, the process temperature, but also relies on the physico-chemical characteristics of crop material and process liquids [7]. Once the biofilm is established, it functions as a cooperative consortium leading to enhanced biomass degradation and, in consequence, to biogas production [8, 9].

Comprehensive microbiome analyses by means of metagenome sequencing revealed that many biogas microbiome members could not be classified down to the species level and hence are currently unknown (‘microbial dark matter’) [10, 11]. As example published previously, in the metagenome dataset determined for a biogas plant operated under thermophilic temperature regime, only 18% of the included 16S rRNA gene sequences were assignable to a taxonomically established genus [10]. In addition, information on the metabolic activity of biogas biofilm microbial communities is only rudimentarily available. Most of the corresponding studies focus on the methanogenic sub-community, while the metabolic potential of the entire biogas biofilm microbiome remains poorly characterized [12, 13]. For this propose, the integration of different -*omics* approaches (integrated or poly-*omics*), as example, the combination of metagenome with metatranscriptome datasets, is indispensable to distinguish between metabolically active and less active microbial species. Genome-centered biogas microbiome analyses already disclosed and prospectively will disclose further functionalities and interactions of keystone microbiome members [14–21].

The degradation of plant biomass by hydrolytic enzymes of surface attached cellulolytic and hydrolytic bacteria and the subsequent secondary fermentation still represent bottlenecks in the engineered optimization of biogas processes. For economically optimal AD process operation, cellulolytic / hydrolytic biofilms adapted to maximal OLRs and, with special respect to the fermentation of substrates with high contents of nitrogen-containing compounds such as proteins and peptides (e.g., grass silage), microbiomes featuring tolerance to high ammonium / ammonia contents are essentially required. It was hypothesized that such biofilm members, in particular functional keystone species, will become recognizable by their increasing abundances and transcriptional activities under demanding process conditions.

**Fig. 1:**
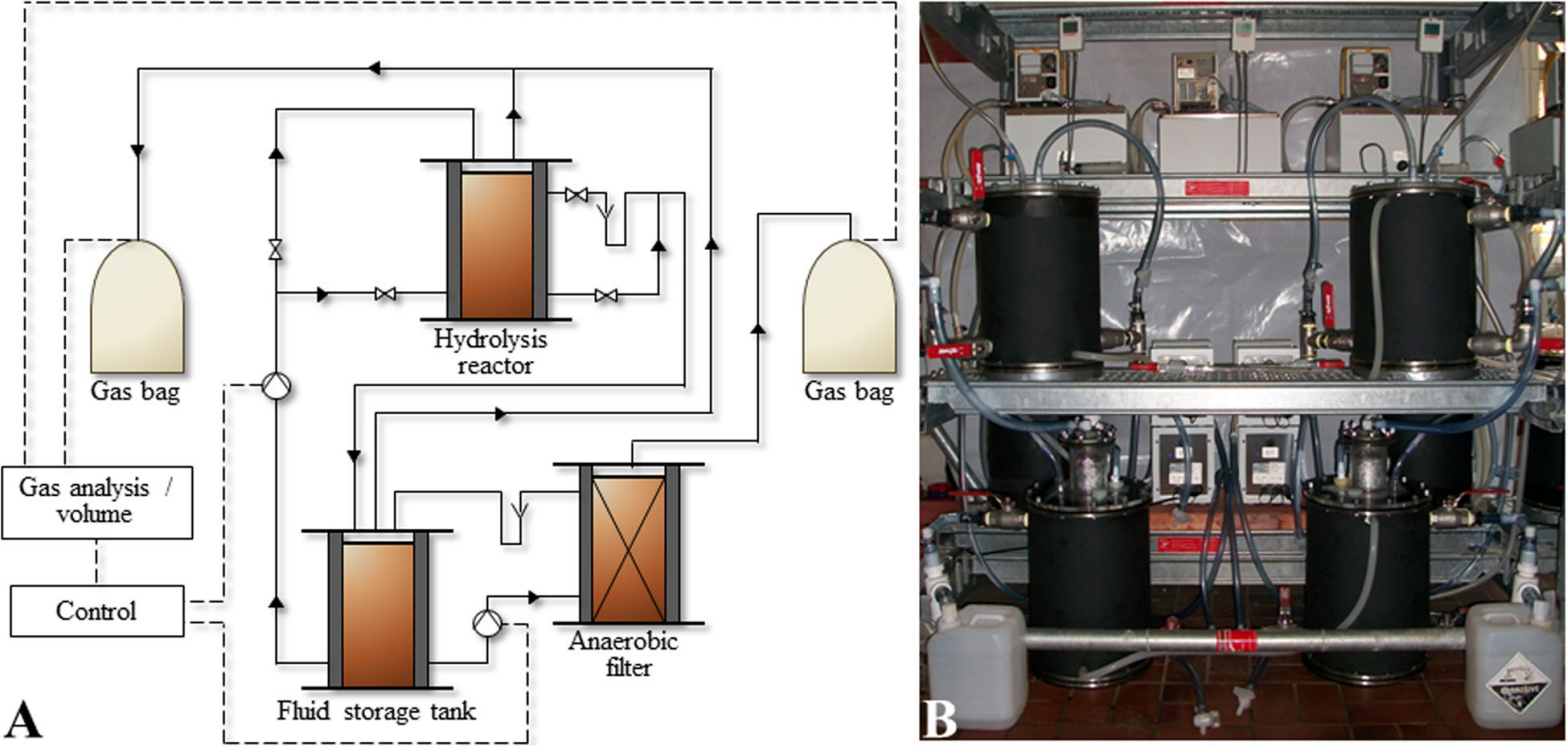
Flow scheme (**a**) and picture (**b**) of the two-stage two-phase biogas reactor system

To unravel the structure, functionality, and metabolic activity of such cellulolytic / hydrolytic biofilm microbiomes, in this study, an integrated -*omics* approach was applied consisting of parallel microbial metagenome and metatranscriptome analyses. To obtain direct access to these microbiomes, biofilm samples grown on the surface of ryegrass silage digested in the hydrolysis reactors (HR) of two-stage two-phase biogas reactor systems (Fig. 1) operated under mesophilic (37 °C) and thermophilic (55 °C) temperature regime and at two (low and increased) OLRs were analyzed. Metabolic reconstruction for candidate metagenome-assembled genomes (MAGs) and corresponding genome-centered transcriptome analyses provided insights into life-styles and activities of adapted species.

## Results

### Biogas reactor performance and long-term microbial dynamics

Four biogas reactor systems were operated in parallel over a period of longer than 750 days resulting in two (biological) replicates for mesophilic (M1, M2) and two replicates for thermophilic conditions (T1, T2) (Fig. 1). Sampling of substrate surface attached biofilms was conducted for two different OLRs, i.e., 500 g (low OLR) and 1500 g (increased OLR) of perennial ryegrass silage.

**Fig. 2:**
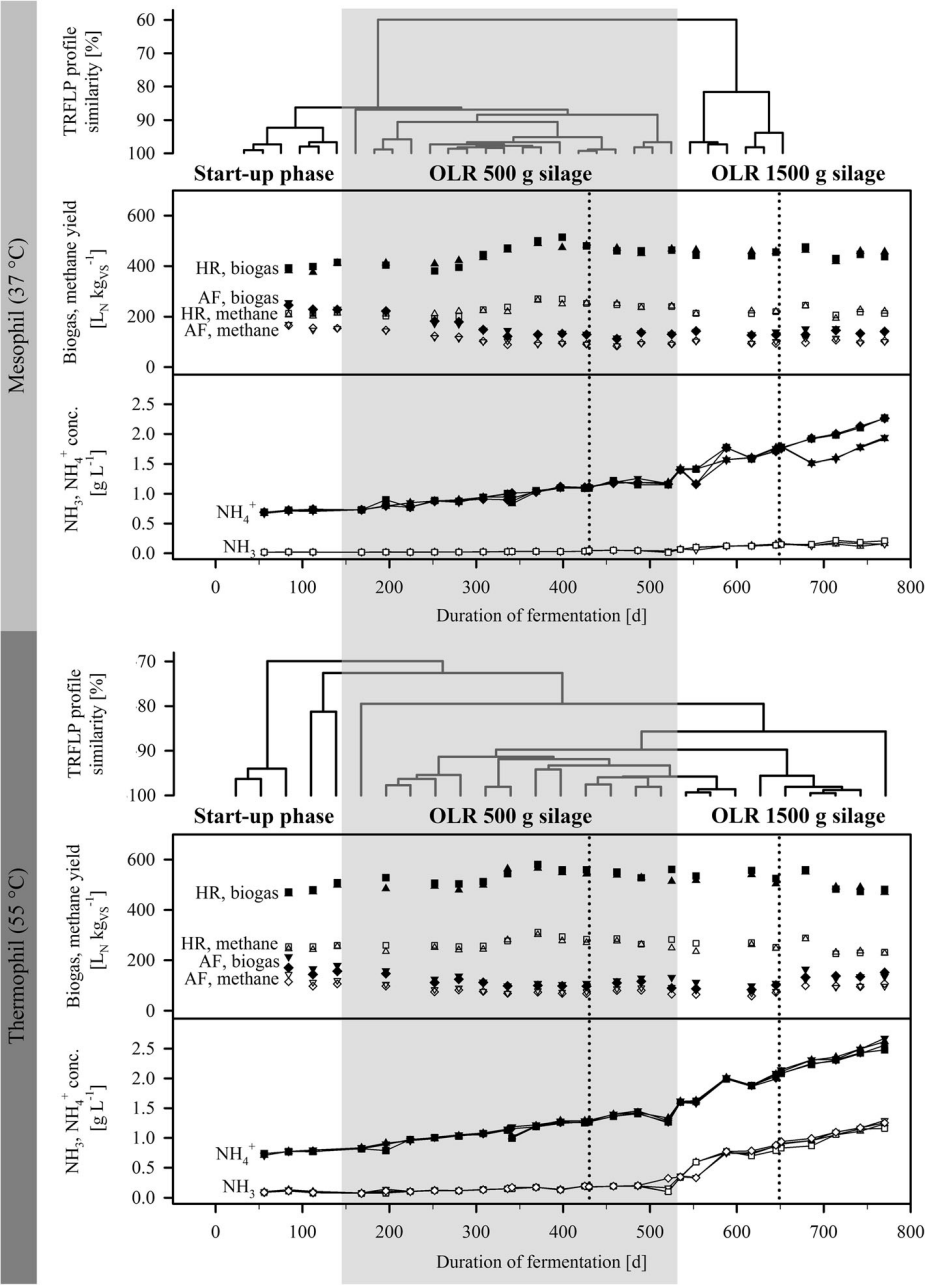
Biogas and methane yields, and NH_4_^+^- and NH_3_-contents in the biogas reactor effluents. Bacterial 16S rRNA gene targeting TRFLP analyses results are shown exemplary for different time points at different process conditions. Dotted lines indicate time points of sampling for NGS. ▲,△, hydrolysis reactor (HR) of reactor system M1 (mesophil) resp. T1 (thermophil);▼, ▽, downstream AF of reactor system M1 (mesophil) resp. T1 (thermophil); ■, □, HR of reactor system M2 (mesophil) resp. T2 (thermophil); ◆, ◇, AF of reactor system M2 (mesophil) resp. T2 (thermophil); L_N_, liters normalized to 0 °C and 1013 hPa; VS, volatile substances; OLR, organic loading rate

The summarized average biogas yields from hydrolytic reactors (HR) and anaerobic filters (AF) were constantly 601 ± 18 l normalized for standard temperature and pressure (L_N_) per kg volatile substances (VS) (M1) and 599 ± 26 L_N_ kg_VS_^− 1^ (M2) for the reactor systems operated under mesophilic temperature regime with an average methane content of 56 ± 2% (v/v) (Fig. 2). Under the thermophilic temperature regime, the summarized systems’ biogas yields were slightly higher with average values of 645 ± 27 L_N_ kg_VS_^− 1^ (T1) and 644 ± 19 L_N_ kg_VS_^− 1^ (T2) with a slightly lower average methane content of 54 ± 2% (v/v) each. The average methane yields ranged from 337 ± 20 L_N_ kg_VS_^− 1^ (M2) to 348 ± 19 L_N_ kg_VS_^− 1^ (T2) which is less than 5% lower than the reference value of, on average, 353 L_N_ kg_VS_^− 1^ as determined by standard batch fermentation tests according to the German technical standard VDI 4630. Even if, in general, the methane content in the biogas was higher in the AF than in the HR (mesophilic, 71 ± 3% *vs*. 51 ± 2%; thermophilic, 70 ± 2% *vs*. 50 ± 2%), most of the biogas and methane was produced in the HR (mesophilic, 74 ± 6% and 67 ± 6% on average; thermophilic, 79 ± 4% to 81 ± 4% and 73 ± 4% to 76 ± 4% depending on the respective process status).

The main product of bacterial fermentation in the HR was acetic acid. Highest concentrations were determined for fermentation day 2 with values ranging from 0.80 g L^− 1^ (start-up phase) to 5.30 g L^− 1^ (OLR 1500 g silage) and under the thermophilic conditions, from 0.91 g L^− 1^ (start-up phase) to 4.21 g L^− 1^ (OLR 1500 g silage). Propionic acid was produced only in minor amounts of up to 0.58 g L^− 1^ (mesophilic) and 0.43 g L^− 1^ (thermophilic). The volatile fatty acids (VFA) accumulation throughout the ongoing fermentation process was not observed.

Due to the nitrogen content of the silage of 7.6 g kg_FM_^− 1^, a NH_4_^+^ accumulation of up to 2.3 g L^− 1^ (mesophilic) and 2.7 g L^− 1^ (thermophilic) was observed in the process fluids during the fermentation and within the entire biogas reactor system (Fig. 2). In contrast to the fermentations at mesophilic temperatures, under thermophilic temperature regime also an accumulation of cytotoxic NH_3_ of up to 1.3 g L^− 1^ occurred.

As revealed by bacterial 16S rRNA gene targeting TRFLP analysis, the bacterial community structure had adapted during the ongoing biomethanation experiment (Fig. 2). It is assumed that adaptation of the community primarily occurred as response to the increase in OLR. Likewise, also the increase in NH_4_^+^ concentrations and, in particular during the thermophilic fermentations, the increase in NH_3_ concentrations may had affected the community composition.

### Cellulolytic / hydrolytic biofilm community structure

To characterize the structure of the bacterial biofilms established on the surface of ryegrass silage digestate in the mesophilic and thermophilic HRs, next generation sequencing (NGS) of the 16S rRNA gene was performed. Due to the used primer sets, primarily 16S rRNA genes for the domain *Bacteria* were detected. 16S rRNA gene sequences for methanogenic *Archaea* were only detected for mesophilic biofilm samples (in maximum, with an abundance of 1.2% in case of the HR biofilm sample M1_OLR1500_) and assigned to genus *Methanosaeta,* i.e.*, Methanothrix*.

Overall, 66% of the 16S rRNA gene sequences were classified into operational taxonomic units (OTUs) that could be assigned to known genera. 34% remained unassigned at genus rank (Additional file 1). In general, the thermophilic biofilms exhibited a slightly lower diversity than the mesophilic biofilms (Shannon indices in average 2.9 vs. 3.2).

**Fig. 3:**
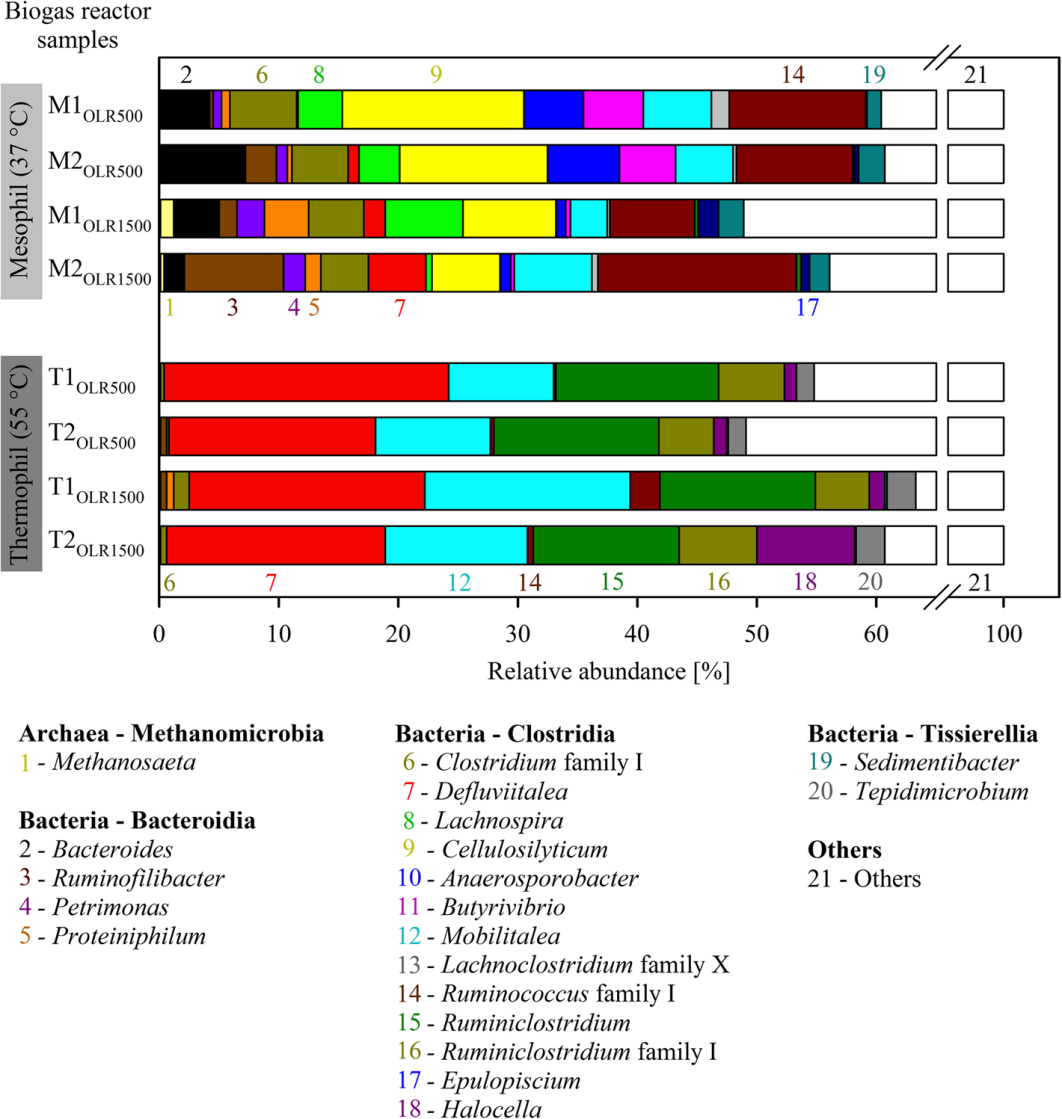
Taxonomic profiling of the hydrolysis reactor (HR) biofilm microbiome. Relative abundances are shown for the most abundant genera of microbial sub-communities as determined by 16S rRNA gene amplicon NGS. M1, M2, mesophilic replicates; T1, T2, thermophilic replicates; OLR500, OLR1500, organic loading rates of 500 or 1500 g ryegrass silage. For further details on sample denomination, refer to Fig. 2

In the mesophilic biofilms, a number of genera were exclusively detected belonging to the classes *Clostridia* (i.e., *Cellulosilyticum*, *Lachnospira*, *Anaerosporobacter*, *Butyrivibrio*, and *Epulopiscium*), *Bacteroidia* (i.e., *Bacteroides*, and *Petrimonas*), *Tissierellia* (i.e., *Sedimentibacter*) (Fig. 3, Additional file 2). In general, the abundance of particular OTUs varied between the biological replicates and OLRs indicating varying microbiome structure.

In the thermophilic biofilms, members of the genera *Ruminiclostridium* and *Halocella* (phylum *Firmicutes*, class *Clostridia*), and *Tepidimicrobium* (class *Tissierellia*) were found, which were not or only detected in minor amounts in the mesophilic biofilms. Also in these biofilms, the abundance of OTUs varied between biological replicates and OLRs. Some genera were present in all biofilms, such as *Ruminococcus*, *Defluviitalea*, and *Mobilitalea* (class *Clostridia*), but all showing substantial differences in abundance in mesophilic and thermophilic biofilms (Fig. 3, Additional file 2).

Under mesophilic temperature regime, the increase in OLR had a positive effect on the abundance of some members, e.g., of the genera *Proteiniphilum* (OLR 500 g: 0.7 resp. 0.4% vs. OLR 1500 g: 3.7 resp. 1.3%) and *Defluviitalea* (OLR 500 g: 0.1 resp. 0.9% vs. OLR 1500 g: 1.8 resp. 4.8%). Other biofilm members decreased in abundance after increase of OLR, such as members of genera *Anaerosporobacter* (OLR 500 g: 5.0 resp. 6.0% vs. OLR 1500 g: 0.8 resp. 0.9%) and *Cellulosilyticum* (OLR 500 g: 15.2 resp. 12.4% vs. OLR 1500 g: 7.8 resp. 5.7%) (Additional file 2).

At thermophilic temperatures, only the genus *Mobilitalea* benefitted from the increased OLR (OLR 500 g: 8.8 resp. 9.6% vs. OLR 1500 g: 17.2 resp. 11.9%) (Additional file 2). All other genera remained at more or less similar abundances. Thus, it can be assumed that *Mobilitalea* species are more tolerant towards increased NH_3_ concentrations compared to other species.

### MAG abundance in the cellulolytic/hydrolytic biofilms depending on the temperature and the OLR

To determine and characterize differential abundances of species represented by metagenomically assembled genomes (MAGs) in the cellulolytic/hydrolytic biofilms established on the surface of ryegrass silage, microbial metagenome sequencing followed by a combined assembly of sequence data and genome binning were applied (Additional file 3). In total, 157 MAGs were compiled and taxonomically classified (Additional file 4). 74 MAGs were assigned to the domain *Bacteria*, five MAGs to *Archaea*, and 78 remained unclassified at domain level. At the phylum level, the MAGs were allocated to the *Firmicutes* (55), *Bacteroidota* (12), *Euryarchaeota* (5), *Spirochaetota* (2), *Fibrobacterota* (1), and *Cloacimonadota* (1). At the family level, only 53 MAGs were classifiable among others to the *Lachnospiraceae* (19), *Bacteroidaceae* (4), *Ruminoclostridiaceae* (3), *Clostridiaceae* (3), indicating occurrence of so far unknown microbial species and/or insufficient representation of adequate reference genomes in databases that are available for comparative analyses.

To uncover the relative abundances of the compiled MAGs in the HRs analyzed, metagenome sequences obtained from the mesophilic and thermophilic microbial communities were mapped onto the MAG sequences. Only 78 MAGs featuring contamination rates below 10% were considered for this approach. Deeper metagenome sequencing would certainly have improved the completeness and number of compiled MAGs. However, sequencing depth always is a trade-off between incurred costs and expected results.

**Fig. 4:**
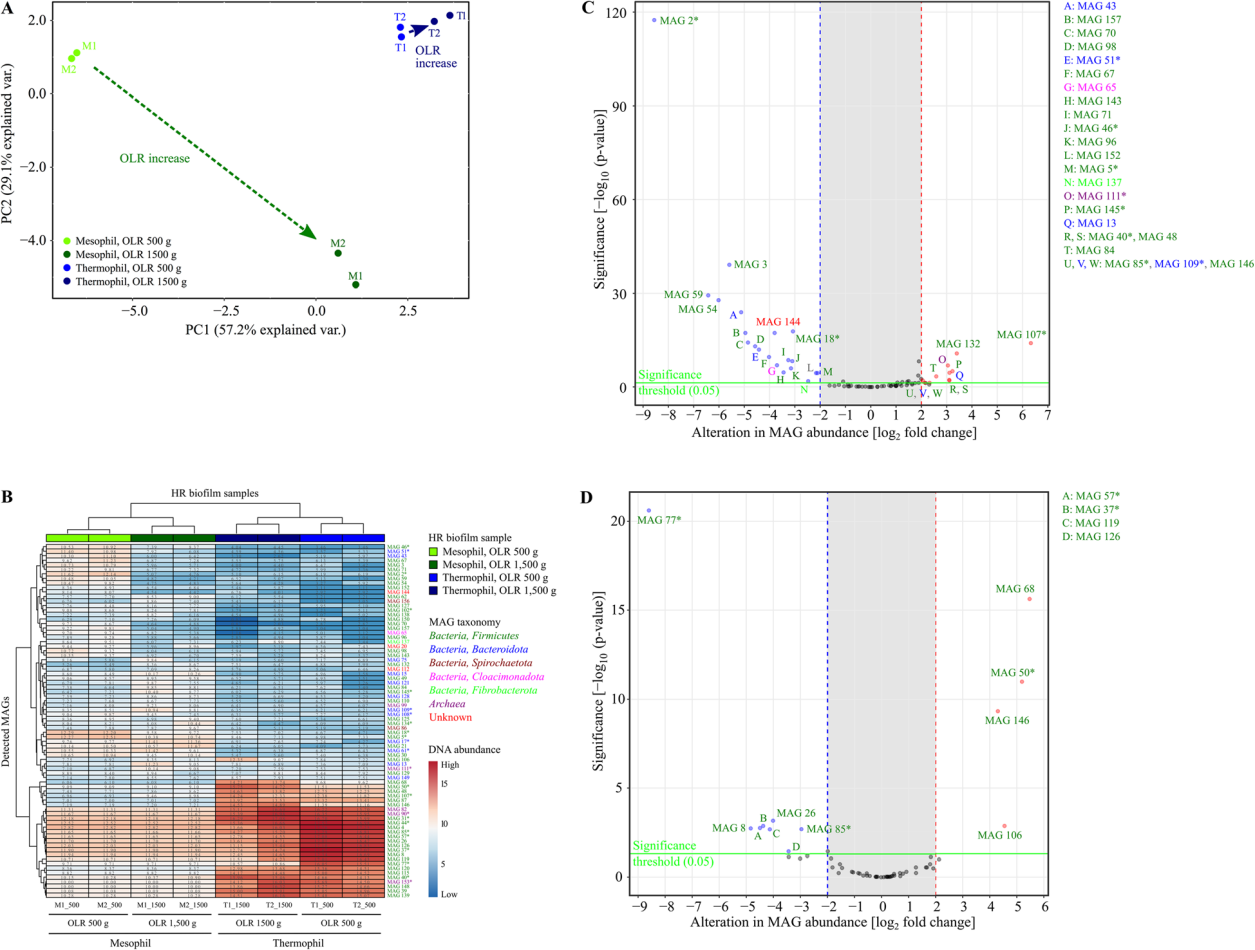
Alteration of abundance of 157 metagenome-assembled genomes (MAGs) detected in HR biofilms at mesophilic (M) and thermophilic (T) process temperature in response to the increase of organic loading rate (OLR) from 500 to 1500 g ryegrass silage as deduced from metagenome data. PCA plots are shown for principle component analyses of scaled and centered *rlog* transformed metagenome read counts mapped on MAGs (**a**). The hierarchical clustering of rlog transformed abundance values for 78 (contamination below 10%) selected MAGs detected in HR biofilms is visualized in (**b**). Green, mesophilic HR biofilms; blue, thermophilic HR biofilms. For further details on sample denomination, refer to Figs. 2 and 3. Alteration in abundance values of 78 MAGs selected in response of the increase of OLR from 500 to 1500 g ryegrass silage at mesophilic (**c**) and thermophilic (**d**) process temperature. Colors of the MAGs indicate taxonomic affiliation (green, *Firmicutes*; blue, *Bacteroidota*; violet, *Archaea*; pink, *Cloacimonadota*; light green, *Fibrobacterota*; dark red, *Spirochaetota*; red, unknown affiliation). * indicate MAGs with completeness above 50% and contamination rate less than 10% as listed in Table 1 and in Additional file 4

Principal component (PCA) analysis of MAG abundance profiles revealed close clustering of replicates confirming reproducibility of the treatments carried out (Fig. 4a). Moreover, temperatures (mesophilic *vs*. thermophilic) led to a clear separation of MAG abundance profiles in the PC plots. Likewise, the OLRs (OLR500 vs. OLR1500) differentiate MAG abundances. However, this effect is far more pronounced for the mesophilic systems as compared to the thermophilic ones (Fig. 4a).

MAG abundance profiles are visualized for all conditions tested (two temperatures and two OLRs in replicates) in heat-maps for the 78 MAGs tested (Fig. 4b). Cluster analysis revealed that the temperature is the most important factor that drives shaping of the community followed by the OLR. Replicates are very similar to each other regarding MAG abundance profiles under the conditions tested.

Relative abundances of the following MAGs significantly increased (log_2_ fold-change of > 2 and –log_10_(*p*-value) of > 0.05) under mesophilic conditions when the OLR was raised to 1500 g: MAG 13, 40, 48, 84, 85, 107, 109, 111, 132, 145 and 146 as shown by volcano plot analysis (Fig. 4c). Relative abundances of several more MAGs decreased under high OLRs. Under thermophilic conditions, the MAGs 50, 68, 106 and 146 increased in abundance upon OLR raise (Fig. 4d). Responsive MAGs featuring completeness value of more than 50% and contamination less than 10% were further analyzed regarding their genetic potential and transcriptional activity. These are MAGs 40, 50, 85, 107, 109, 111 and 145 (see below).

### Functional potential of MAGs positively responding to increased OLR

**Fig. 5:**
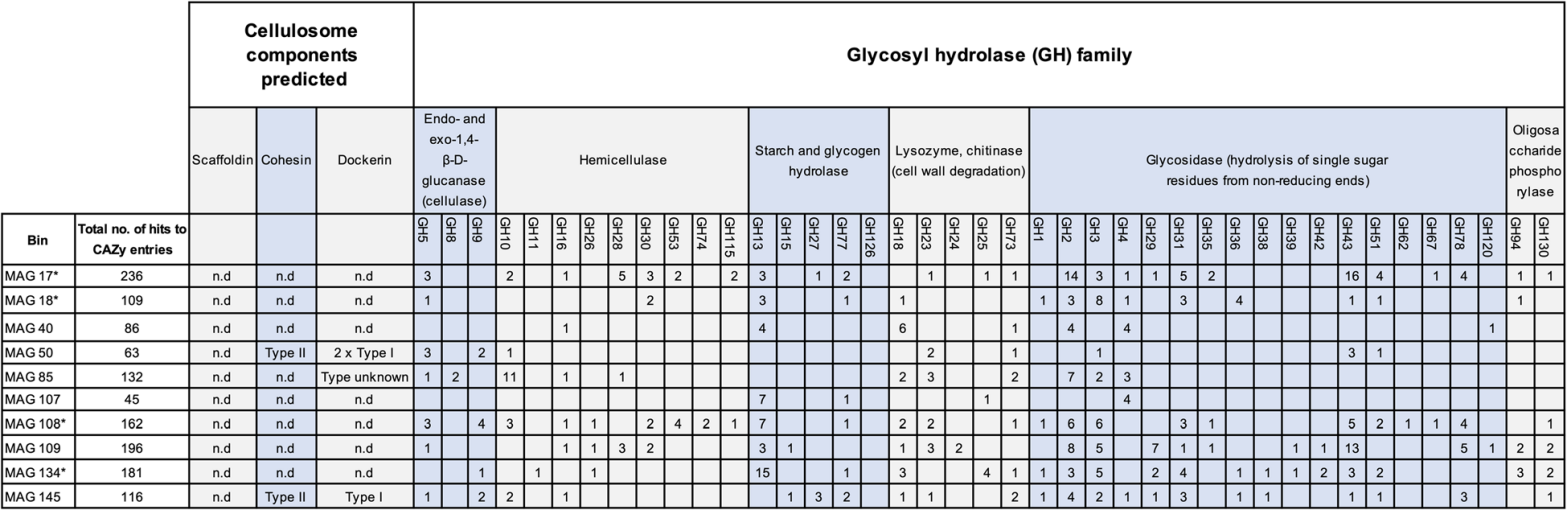
Genes encoding carbohydrate-active enzymes predicted for bacterial metagenome-assembled genomes (MAGs) most abundant in hydrolysis reactor (HR) biofilms. * These MAGs showed increased transcriptional activity in response to the organic loading rate and were therefore additionally analyzed

To gain insights into the functional potential of MAGs positively responding to increased OLRs, genetic determinants for utilization of carbohydrates (Fig. 5) as well as the key enzymes of AD pathways were analyzed (Additional file 5). The genetic determinants were categorized according to the four stages of the AD process, namely hydrolysis, acidogenesis, acetogenesis and methanogenesis as described previously [23].

Regarding the functional context ‘hydrolysis’, the genetic potential for utilization of carbohydrates was characterized in the selected bacterial MAGs. Genes encoding carbohydrate-active enzymes were determined by applying the HMM-based Carbohydrate-active enzyme annotation database dbCAN v7 [24] (Fig. 5). Between 45 and 236 genes encoding cellulosomal proteins or enzymes with predicted activities on carbohydrates were identified in each of the bacterial MAGs analyzed.

Obtained results subdivided the analyzed MAGs into two groups. Group I members were predicted to encode cellulosome structures required for efficient degradation of cellulose, comprising dockerin-containing glycosyl hydrolases (GHs), corresponding cohesin-containing scaffoldins, and enzymes acting on large carbohydrate molecules. Some of the identified enzymes contain carbohydrate-binding motifs. MAG 50 (family *Defluviitaleaceae*), MAG 85 (phylum *Firmicutes*) and 145 (affiliated to the genus *Jeotgalibaca*) represent group I members. In general, for cellulolytic/hydrolytic biofilms, the MAGs belonging to group I are of great importance, since they represent bacterial candidates featuring the potential for efficient decomposition of complex carbohydrates such as cellulose, hemicellulose and xylan.

The remaining MAGs were classified to group II representing secondary fermentative bacteria mainly utilizing mono-, di- and oligosaccharides for energy production as supplied by group I bacteria. Group II comprises MAGs lacking genes for proteins involved in cellulosome formation and assembly. However, due to the incompleteness of these MAGs (Table 1), it cannot be excluded that cellulosome genes were missed during the assembly and binning processes.

**Table 1:** Taxonomic affiliations and characteristics of selected metagenome-assembled genomes (MAGs) most abundant in HR biofilms

MAG	Taxonomic affiliation^1^					MAG features						
	Phylum	Class	Order	Family	*Genus*	Comleteness^2^ [%]	Contamination^2^ [%]	Size [bp]	GC content [%]	No. of genes	No. of rrn genes	No. of tRNA genes
*Bacteria*												
13	*Firmicutes*	Unknown	Unknown	Unknown	Unknown	29.74	3.43	1,239,774	44.81	1159	3	31
17*	*Bacteroidetes*	*Bacteroidia*	*Bacteroidales*	*Bacteroidaceae*	*Prevotella* sp.	68.02	8.98	2,689,597	48.90	2294	2	39
18*	*Firmicutes*	*Clostridia*	*Lachnospirales*	*Lachnospiraceae*	*Acetivibrio* sp.	68.50	3.65	2,855,089	39.03	2917	n/d^3^	15
40*	*Firmicutes*	*Clostridia*	*Lachnospirales*	*Lachnospiraceae*	*Herbinix* sp.	68.42	7.66	1,849,940	36.65	1755	n/d	8
48	*Firmicutes*	*Clostridia*	*Lachnospirales*	*Lachnospiraceae*	*Herbinix* sp.	26.48	2.63	770,644	37.4	614	n/d	3
50	*Firmicutes*	*Clostridia*	*Lachnospirales*	*Defluviitaleaceae*	Unknown	51.74	6.14	1,725,349	31.02	1775	n/d	31
68	*Firmicutes*	*Clostridia*	*Lachnospirales*	*Lachnospiraceae*	*Herbinix* sp.	52.17	4.77	2,358,488	46.61	2310	2	27
84	*Firmicutes*	*Clostridia*	*Lachnospirales*	Unknown	Unknown	35.22	5.14	1,970,476	38.81	1672	2	17
85	*Firmicutes*	Unknown	Unknown	Unknown	Unknown	80.11	3.08	2,011,311	47.98	2076	n/d	33
106	*Firmicutes*	*Clostridia*	*Clostridiales*	*Clostridiaceae*	*Clostridium* sp.	33.09	4.39	883,787	31.79	812	n/d	17
107*	*Firmicutes*	*Clostridia*	*Clostridiales*	*Clostridiaceae*	*Clostridium* sp.	50.06	6.97	1,539,556	29.37	1648	n/d	17
108*	*Bacteroidetes*	*Bacteroidia*	*Bacteroidales*	Unknown	Unknown	79.01	8.21	2,032,215	47.60	2025	1	29
109	*Bacteroidetes*	*Bacteroidia*	*Bacteroidales*	*Dysgonomonadaceae*	*Proteiniphilum* sp.	63.52	7.19	2196,928	45.07	2196	1	27
132	*Firmicutes*	*Clostridia*	Unknown	Unknown	Unknown	26.26	2.37	1,284,977	37.03	1286	n/d	16
134*	*Firmicutes*	*Clostridia*	*Lachnospirales*	*Lachnospiraceae*	*Kineothrix* sp.	78.46	9.27	2,788,291	40.86	2946	n/d	31
145	*Firmicutes*	*Bacilli*	*Lactobacillales*	*Aerococcaceae*	*Jeotgalibaca* sp.	63.37	9.48	2,346,037	46.04	2647	n/d	14
146	*Firmicutes*	*Bacilli*	Unknown	Unknown	Unknown	33.22	0.00	861,844	38.49	705	1	13
Archaea												
111	*Euryarchaeota*	*Methanobacteria*	*Methanobacteriales*	*Methanobacteriaceae*	*Methanobacterium* sp.	73.74	8.57	2,134,563	37.98	2406	2	51

^1^ GTDB-Tk based classification

^2^ For details, refer to [22] and Additional file 4

^3^ n/d = not detected

* MAGs which showed increased transcriptional activity in responce to the organic loading rate (OLR)

Furthermore, genetic determinants encoding key enzymes required for utilization of different organic molecules such as pyruvate, lactate, ethanol, acetate, propionate and butyrate representing important metabolites of the acidogenesis and acetogenesis were analyzed in the MAGs selected (Additional file 5). As examples, the bacterial MAGs 109 (*Proteiniphilum* sp.) and 145 (*Jeotgalibaca* sp*.*) encode high numbers of key genes featuring predicted functions in pyruvate metabolism (between 14 and 21), also representing the KEGG (map 00620) modules for utilization of lactate (between 5 and 7) and acetate (8) only in case of the MAG 145. In MAG 145, essential genes encoding enzymes of the Wood-Ljungdahl pathway (8) were identified, which plays an important role in carbon fixation and acetate utilization.

During acetogenesis, several bacterial species utilize propionic acid employing the methylmalonyl-CoA or the acrylyl-CoA pathway of the propanoate metabolism. The bacterial MAGs 109 (*Proteiniphilum* sp.) and 145 (*Jeotgalibaca *sp.) possess several genes (between 5 and 9) for enzymes that were assigned to the methylmalonyl-CoA branch of the propanoate metabolism. Furthermore, MAGs 109 and 145 additionally encode the methylmalonyl-CoA mutase Mut (EC 5.4.99.2) and the methylmalonyl-CoA/ethylmalonyl-CoA epimerase (EC 5.1.99.1) representing the key enzymes of this metabolism as described by Sikore et al. [23]. Therefore, these MAGs were predicted to be involved in the propionic acid metabolism in the analyzed cellulolytic/hydrolytic biofilms. Moreover, MAGs 50, 85, 109 and 145 most probably are involved in butanoate metabolism since they possess between 2 and 6 of key genes classified to the butanoate pathway (KEGG map00650). The butanoate pathway is not completely encoded in the MAGs analyzed. However, genes encoding relevant key enzymes described by Sikora et al. [23] were identified in the genomes of these *Bacteria*. The MAG 109 harbors a gene encoding PFL-pyruvate formate lyase (EC 2.3.1.54), whereas MAG 145 possesses the gene encoding butyrate kinase (EC 2.7.2.7) indicating the importance of these bacteria for AD at mesophilic temperatures.

The formation of methane, the last step of AD, is performed by members of the phylum *Euryarchaeota*. In the analyzed biofilms, MAG 111 (genus *Methanobacterium* sp.) was detected as most abundant archaeon positively responding to the increase in OLRs. Twenty-two genes encoding key enzymes of the hydrogenotrophic methanogenesis pathway were identified in this MAG illustrating the importance of this pathway under increased OLRs.

### Transcriptional activity of MAGs in response to temperature and OLR

To determine the transcriptional activities of the compiled MAGs, the metatranscriptomes from the different reactor systems were sequenced capturing the prevailing conditions (temperature: mesophilic vs. thermophilic, OLRs of 500 g vs. 1500 g). Mapping of the transcriptome sequences for each dataset to the assembled contigs allowed determination of the MAG’s transcriptional activities under the conditions tested.

**Fig. 6:**
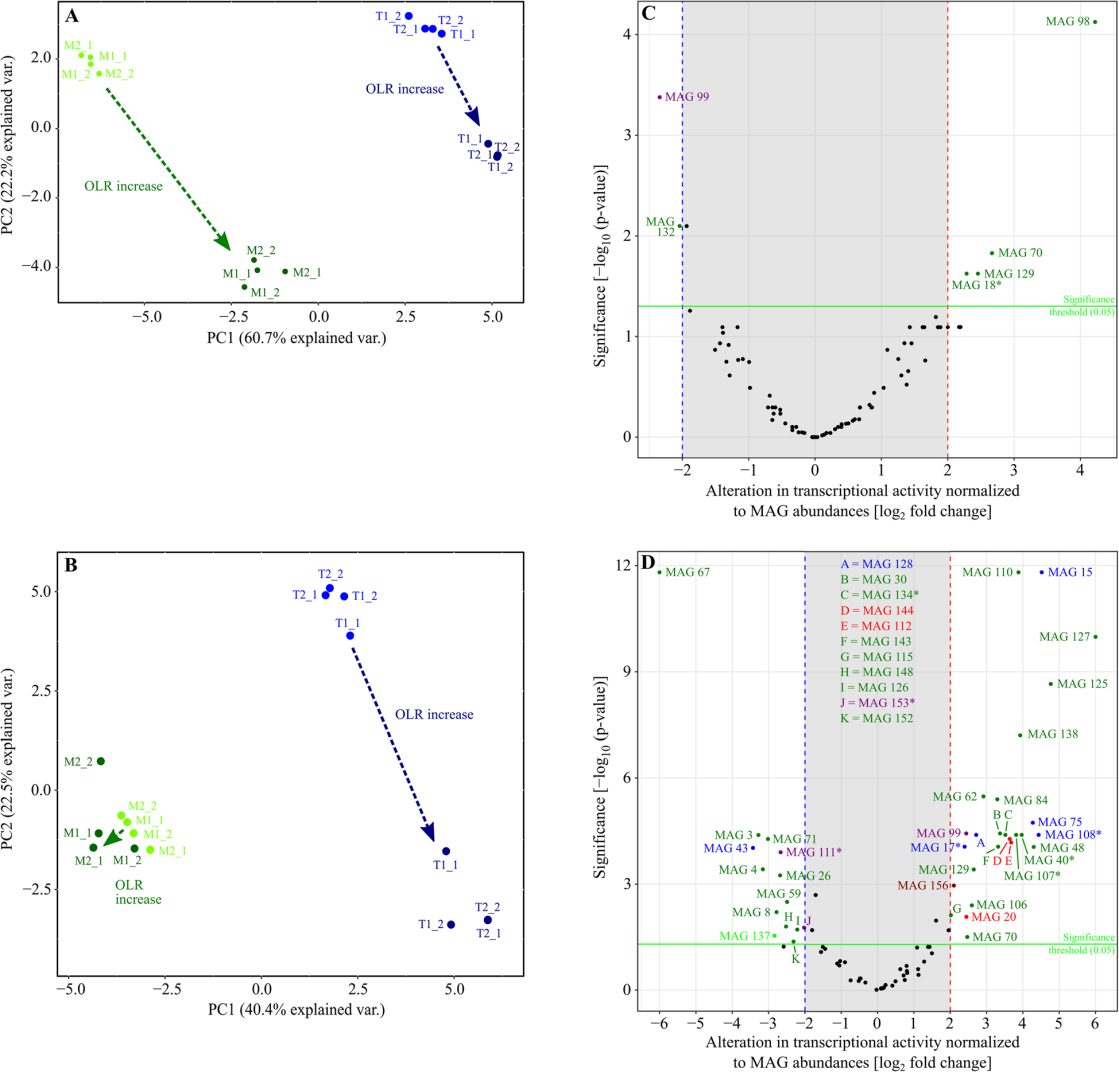
Alteration in transcriptional activity of 78 selected metagenome-assembled genomes (MAGs) detected in HR biofilms at mesophilic (M) and thermophilic (T) process temperature in response of the increase of organic loading rate (OLR) from 500 to 1500 g ryegrass silage. PCA plots are shown for principle component analyses of scaled and centered *rlog* transformed metatrabscriptome read counts mapped on MAGs (**a**). Transcripts were normalized on the total number of sequencing reads, assuming equal MAG abundance, and were averaged for biological replicates (**b**). Green, mesophilic HR biofilms; blue, thermophilic HR biofilms. For further details on sample denomination, refer to Figs. 2 and 3. Alteration in transcript abundance of MAGs selected in response of the increase of OLR from 500 to 1500 g ryegrass silage at mesophilic (**c**) and thermophilic (**d**) process temperature. Colors of the MAGs indicate taxonomic affiliation (green, *Firmicutes*; blue, *Bacteroidota*; violet, *Archaea*; pink, *Cloacimonadota*; light green, *Fibrobacterota*; dark red, *Spirochaetota*; red, unknown affiliation). * indicate MAGs with completeness above 50% and contamination rate less than 10% as listed in Table 1

Principal component (PCA) analysis revealed clear separation of the MAG’s transcriptional activity patterns in relation to the temperature (mesophilic and thermophilic) and the OLRs 500 g and 1500 g (Fig. 6a). However, compared to the corresponding analysis based on MAG abundance profiles (see Fig. 4a), the thermophilic groups representing OLR500 and OLR1500 are more distinct suggesting transcriptional differences between these groups leading to the observed separation of the respective patterns. Clustering of transcriptional activity patterns yielded two main clusters representing the mesophilic and the thermophilic temperature regime and further sub-clusters representing the different OLRs (OLR500 and OLR1500) for each reactor system (Additional file 6). The transcriptional activities of the MAGs 40 (order *Lachnospirales*, *Herbinix* sp.), 48 (order *Lachnospirales*, *Herbinix* sp.), 107 (order *Clostridiales*, *Clostridium* sp.), 111 (phylum *Euryarchaeota*, *Methanobacterium* sp.) and 132 (class *Clostridia*, unknown species) increased in response to the OLR1500 in the mesophilic reactors. These MAGs also showed a positive response upon OLR increase in the abundance analysis (Fig. 4b). The response of further MAGs displayed in Additional file 6 is significant but not as pronounced as compared to the aforementioned ones. Likewise, MAGs 50 (class *Clostridia*, unknown species), 68 (class *Clostridia*, *Herbinix* sp.), 106 (class *Clostridia, Clostridium* sp*.*) and 146 (class *Bacilli*, unknown species) respond to the increased OLR with higher transcriptional activities in the thermophilic reactors (Additional file 6). These observations may be explained by the obvious connection between transcriptional activity increase and proliferation enhancement. However, for the thermophilic reactor systems, there are 14 more MAGs significantly responding to the higher OLR1500 by increasing their transcriptional activities (Additional file 6). However, is should be noted that these MAGs were not apparent in the Volcano plot based on MAG abundance profiles (Fig. 4c-d).

### Transcriptional activity of MAGs normalized to MAG abundances

In order to investigate the transcriptional activity of MAGs regardless of their abundances, transcriptome read counts for MAGs were also normalized according to MAG abundances (for details refer Material and Methods). Examination of resulting normalized transcriptional rates by applying PCA revealed well separated groups representing the different OLRs (OLR500 and OLR1500) only for the thermophilic reactor systems (Fig. 6b). This observation indicates that under thermophilic conditions, particular MAGs are able to respond to the OLR by shifting their transcriptional activity without simultaneously adjusting their replication rates. Under mesophilic conditions, this behavior only is of minor importance. MAGs responding to the higher OLR with increased normalized transcription rates are depicted in Fig. 6c-d. These are the MAG 18 (order *Lachnospirales*, *Acetivibrio* sp.) originating from the mesophilic biofilm as well as MAGs 17 (order *Bacteriodales*, *Prevotella* sp.), 40 (order *Lachnospirales*, *Herbinix* sp.), 107 (order *Clostridiales*, *Clostridium* sp.), 108 (order *Bacteroidales*, unknown species) and 134 (order *Lachnospirales*, *Kineothrix* sp.) obtained from the thermophilic HRs.

In summary, under thermophilic conditions, several MAGs feature an additional transcriptional enhancement that is decoupled from their replication rates which may be explained by increased metabolic activity at higher (thermophilic) temperatures.

### Transcriptional profiling of most active cellulolytic / hydrolytic biofilm MAGs

To study the transcriptional activities related to OLRs in more detail, MAG 18 for the mesophilic biofilm as well as MAGs 17, 40, 107, 108 and 134 for the thermophilic biofilm were selected, since they showed the highest transcriptional increases in response to OLR increase (Fig. 6c-d, Table 1). Transcriptional rates represented by TPM values were calculated for all genes in each MAG. Subsequently, lists of the 100 most highly transcribed genes based on their TPM values were compiled for each MAG, additionally specifying the encoded gene products and their predicted functions. Transcripts with TPM values of ≤0.5 were not considered further due to their low significance. Transcripts encoding proteins involved in mandatory functions (basic house-keeping functions) such as transcription and translation were not further considered. The top five transcripts of each MAG as well as remaining genes that are of importance for assessing the metabolism of the organism were further analyzed (see Table 2).

**Table 2:** Most actively transcribed genes for enzymes involved in carbohydrate or protein utilization of selected HR biofilm MAGs

Metagenome assembled genome (MAG)^1^	Assumed taxonomic affiliation	Analyzed HR^2^ biofilm sample	Gene transcripts involved in carbohydrate or protein utilization							Predicted functional role in AD^4^
			GenID	Gene length	Putative gene product	Gene	EC number	Transcripts mapped	Normalized number of transcripts [TPM]^3^	
**17**	*Bacteroidales* (*Prevotella* sp.)	T1_OLR1500_	Bin_17_01477	366	Deoxyguanosinetriphosphate triphosphohydrolase	*dgt*	3.1.5.1	141	3.9	**n/s**
			Bin_17_01805	171	Hypothetical protein	n/s	n/s	28	3.4	
			Bin_17_00602	177	50S ribosomal protein	*rpm*D	n/s	10	1.5	
			Bin_17_00172	651				75	0.8	
			Bin_17_00603	519	50S ribosomal protein	*rps*E	n/s	53	0.8	
			Bin_17_00862	1041	L-3,5-diaminohexanoate dehydrogenase	*kdd*	1.4.1.11	92	0.6	
			Bin_17_00014	579	N-acetylhexosamine 1-kinase	*nah*K	2.7.1.162	38	0.5	
**18**	*Clostridiales* (*Acetivibrio* sp.)	M1_OLR1500_	Bin_18_02374	207	Small; acid-soluble spore protein C2	*ssp*C		4876	619	**Hydrolysis**
			Bin_18_01750	144	Hypothetical protein	n/s	n/s	462	145	
			Bin_18_01164	192	Hypothetical protein	n/s	n/s	384	58	
			Bin_18_00189	786	Sulfurtransferase	*tus*A	2.8.1.-	3938	53	
			Bin_18_01827	108	Hypothetical protein	n/s	n/s	74	46	
			Bin_18_02918	711	Glycerol uptake facilitator protein	*glp*F	n/s	573	9	
			Bin_18_01829	912	Lactose transport system permease protein	*lac*F	n/s	760	9	
			Bin_18_01831	1377	Beta-glucosidase A	*bgl*A	3.2.1.21	798	6	
			Bin_18_01301	1272	Peptidoglycan binding domain protein		n/s	678	5	
			Bin_18_01830	858	L-arabinose transport system permease protein	a*ra*Q	n/s	413	5	
			Bin_18_01016	1119	D-galactose-binding periplasmic protein precursor	*mgl*B	n/s	309	3	
**40**	*Lachnospirales* (*Herbinix* sp.)	M1_OLR1500_	Bin_40_01523	225	Acid-soluble spore protein C2	*ssp*C_1	n/s	42,076	2942	**Hydrolysis**
			Bin_40_01017	114	Manganese containing catalase	n/s	n/s	5156	1711	
			Bin_40_00454	147	Hypothetical protein	n/s	n/s	5508	1017	
			Bin_40_00073	246	Hypothetical protein	n/s	n/s	17,299	1006	
			Bin_40_01138	228	Acid-soluble spore protein C2	*ssp*C_2	n/s	14,627	994	
			Bin_40_00033	1770	Oligopeptide-binding protein	*opp*A	n/s	14,445	50	
			Bin_40_00081	819	Maritimacin	n/s	n/s	4915	42	
			Bin_40_00131	681	Thiamine transporter	*thi*T	n/s	3522	38	
			Bin_40_00519	1116	Trehalose import ATP-binding protein	*sug*C	n/s	6385	37	
			Bin_40_00505	1008	Glyceraldehyde-3-phosphate dehydrogenase	*gapN*	1.2.1.12	4163	27	
			Bin_40_01590	1329	Maltose/maltodextrin-binding protein precursor	*mal*X	n/s	5638	27	
**107**	*Clostridiales* (*Clostridium* sp.)	T2_OLR1500_	Bin_107_01262	183	Small; acid-soluble spore protein C1	*sspC1_1*	n/s	29,669	3262	**Hydrolysis**
			Bin_107_01261	180	Small; acid-soluble spore protein C2	*sspC2_2*	n/s	24,756	2829	
			Bin_107_01260	171	Small; acid-soluble spore protein C2	*sspC2_1*	n/s	16,600	2136	
			Bin_107_00122	186	Small; acid-soluble spore protein C1	*ssp*C1_2	n/s	9026	956	
			Bin_107_00144	234	Glutaredoxin-3	*grxC*	n/s	7124	459	
			Bin_107_01233	258	Peptidase domain protein	n/s	n/s	1245	65	
			Bin_107_00711	99	Enolase	*eno*	4.2.1.11	87	37	
			Bin_107_01338	1530	4-alpha-glucanotransferase	*malQ*	2.4.1.2	4863	20	
			Bin_107_00018	114	L-cystine import ATP-binding protein	*tcyN*	n/s	58	19	
			Bin_107_01344	678	Trehalose import ATP-binding protein	*sugC*	3.6.3.-	1732	19	
			Bin_107_01340	894	Maltose transport system permease protein	*malG*	n/s	2415	19	
			Bin_107_01038	531	PTS system, maltose-specific, EIICB component	*malP*	n/s	1017	16	
			Bin_107_01656	1002	Glyceraldehyde-3-phosphate dehydrogenaseGlyceraldehyde-3-phosphate dehydrogenase	*gapN*	1.2.1.12	2090	14	
			Bin_107_01134	627	Lysozym	*acm*	3.2.1.17	1086	13	
			Bin_107_00091	1881	Amylopullulanase	*apu*	3.2.1.41	3989	13	
			Bin_107_01040	1335	6-phospho-alpha-glucosidase	*pagL*	3.2.1.-	2015	10	
			Bin_107_00188	624	Thiamine transporter	*thiT*	n/s	692	9	
			Bin_107_01547	132	NADP-dependent glyceraldehyde-3-phosphate dehydrogenase	*gap*	1.2.1.9	158	8	
**108**	*Bacteroidales (unclassified genus)*	T2_OLR1500_	Bin_108_01400	213	Hypothetical protein	n/s	n/s	18	1.4	**n/s**
			Bin_108_00987	726	Alkyl hydroperoxide reductase	*ahp*C	1.11.1.15	85	0.8	
			Bin_108_00986	183	Nitrite reductase [NAD(P)H]	*nas*D	1.7.1.4	4	0.4	
			Bin_108_00047	1140	Hypothetical protein	n/s	n/s	60	0.3	
			Bin_108_01184	441	50S ribosomal protein L15	*rpl*O	n/s	14	0.2	
**134**	*Lachnospirales* (*Kineothrix* sp.)	T2_OLR1500_	Bin_134_02838	177	Transition state regulatory protein	*abrB*	n/s	1527	181	**Hydrtolysis**
			Bin_134_00038	555	Hypothetical protein	n/s	n/s	491	7	
			Bin_134_00185	291	ATP-dependent Clp protease, proteolytic subunit	*clpP*	n/s	81	3	
			Bin_134_00184	1278	ATP-dependent Clp protease, ATP-binding subunit	*clpX*	n/s	622	3	
			Bin_134_00305	906	HTH-type transcriptional regulator	*yofA*	n/s	324	2	
			Bin_134_02093	1110	Trehalose import ATP-binding protein	*acm*	3.2.1.17	234	1	
			Bin_134_00012	570	Amylopullulanase	*pul*A	3.2.1.41	74	1	
			Bin_134_00958	3558	Pyruvate-flavodoxin oxidoreductase	*ydbK*	1.2.7.1	623	1	

1 The first five transcripts of each MAG represent the top transcripts of the corresponding MAG

2 Hydrolysis reactor

3 Transcripts per million averaged for datasets from two technical replicates

4 Anaerobic digestion

*n/s* Not specified

For the mesophilic process regime, MAG 18 revealed an increased transcriptional activity depending on the apparent OLR. Genes encoding different carbohydrate transport proteins were found among the most highly transcribed genes. MAG 18 also transcribed the gene *bgl*A encoding an beta-glucosidase (EC 3.2.1.21) acting on terminal, non-reducing beta-D-glucosyl residues resulting in release of beta-D-glucose. Regarding its genome (Fig. 5 and Additional file 5) and transcriptome characteristics, a hydrolytic lifestyle is proposed for MAG 18 which is of particular interest for AD of protein-rich grass silage.

Another thermophilic bacterium showing an increased transcriptional activity in response to the apparent OLR is MAG 40. In particular, MAG 40 potentially is involved in decomposition of di- and monosaccharides trehalose, maltose and glucose. Further highly transcribed genes encode oligopeptide transport system proteins suggesting a hydrolytic lifestyle of this taxon.

MAG 107 is of particular interest since genes for a trehalose import ATB-binding protein of the proposed MalEFG transporter feature high TPM values (see Table 2). In addition, MAG 107 participated in clevage of α-1,6 and α-1,4 glycosidic bonds in branched and linear polysaccharides, producing glucose, maltose and maltotriose as degradation products, since the gene encoding an amylopullulanase PulA (EC 3.2.1.41) was identified among the highly transcribed genes. Its hydrolytic metabolism as well as the high transcriptional activity under elevated OLR and ammonia contents makes MAG 107 (presumably related to *Clostridium* sp.) appear as a potential candidate for further biotechnological improvement of AD of protein-rich silages under thermophilic conditions.

The highest transcribed gene in MAG 134 are the ATP-dependent Clp proteases gene for a component of an oligopeptide membrane transport system, eventually required for protein degradation. Furthermore, within the list of highly transcribed genes, the amylopullulanase (EC 3.2.1.41) gene *pul*A was also identified, whose gene product is involved in cleavage of glycosidic bonds in polysaccharides as described above. Based on these findings, MAG 134 might be involved in oligopeptide and amino acid degradation, and therefore, plays a crucial role in the acidogenesis / acetogenesis process of the biofilm community.

The bacterial MAGs 17 (order *Bacteroidales, Prevotella* sp.) and 108 (order *Bacteroidales*, unknown species) were moderately abundant in thermophilic HR biofilm metagenome datasets but showed significantly increased transcriptional activity when the OLR was raised from 500 to 1500 g (Fig. 6d). However, the transcripts specified by MAG 17 and 108 only contribute to the total transcriptome of the analyzed microbiome to a small extent and therefore these MAGs seem to be of minor importance or only play a secondary role. Accordingly, prediction of the functionality of these MAGs in the hydrolysis of crop biomass remains difficult.

## Discussion

In this study, the cellulolytic/hydrolytic biofilm communities grown on the surface of ryegrass silage digested in mesophilic (37 °C) and thermophilic (55 °C) hydrolysis reactors under low (500 g) and increased (1500 g) OLR conditions were studied. The community profiles were determined by 16S rRNA gene amplicon analysis whereas metagenome sequencing was done to compile metagenomically assembled genomes (MAGs) for abundance estimations and reconstruction of their metabolism. In parallel, the transcriptional activities of MAGs were unraveled by metatranscriptome sequence analysis and genome-centered transcriptome mappings. As result of this study, thermophilic and mesophilic bacterial and archaeal candidate MAGs adapted to high ammonium/ammonia concentrations caused by high substrate protein contents were identified.

The reconstruction of genomes from metagenome datasets using an assembly and binning strategy enabled cultivation-independent recovery of 157 bacterial and archaeal MAGs. These MAGs represent abundant, and therefore important cellulolytic/hydrolytic biofilm community members, with 78 out of 157 MAGs featuring defined quality criteria, necessary for adequate genome sequence analyses.

A global view on the relative abundances of the compiled MAGs in the analyzed HRs revealed clear clustering of MAG abundance profiles in dependence of the temperatures and OLRs (OLR500 vs. OLR1500). Although the temperature is an important factor shaping anaerobic digestion microbiomes, some degree of overlap in taxonomic profiles was observed regarding the mesophilic and thermophilic communities analyzed in the present study. This result is in agreement with previous research since it has been shown that certain taxa are prevalent in mesophilic as well as thermophilic AD microbiomes [25, 26]. This also applies to the methanogenic archaeal sub-community [26, 27]. Therefore, presence of MAG 82 assigned to the *Euryachaeota* in the mesophilic and thermophilic reactor systems of this study is not unusual. The same applies e.g. also to MAG 107 and MAG 119 assigned to the domain *Bacteria*. Certain bacterial species were previously identified under mesophilic as well as thermophilic conditions [28, 29].

The OLR-effect is more pronounced for the mesophilic systems as compared to the thermophilic ones suggesting that under mesophilic conditions, MAGs respond to changes of the OLR by adapting their proliferation rates whereas for the thermophilic MAGs corresponding responses are of minor importance. These analyses allowed identification of mesophilic and thermophilic MAGs positively responding to increased OLRs.

Subsequent analysis of MAG’s transcriptional activities in response to the temperature and OLRs also led to a clear separation of the MAG’s transcriptional activity patterns in response to the conditions applied. As expected, particular MAGs responding to the OLR-increase by proliferation also showed higher transcriptional activities. Interestingly, normalization of transcriptional activity patterns to MAG abundances revealed that under thermophilic conditions, the patterns are clearly separated which is not the case for the mesophilic temperature regime. This result suggests that under thermophilic conditions, specific MAGs mainly respond to the OLR-increase by enhancing or reducing their transcriptional activities without changing their proliferation rates. This is different to the behavior of responsive MAGs under mesophilic conditions (see above). The most outstanding MAGs in this regard were assigned to the order *Lachnospirales* (*Acetivibrio* sp.) for the mesophilic biofilm and the orders *Bacteroidales* (*Prevotella* sp. and an unknown species), *Lachnospirales* (*Herbinix* sp. and *Kineothrix* sp.) and *Clostridiales* (*Clostridium* sp.) for the thermophilic biofilm.

The effects of OLRs on biofilm formation during AD of organic waste were studied previously [9, 30, 31]. However, most of the studies only focused on reactor performance and biofilm dynamics, rather than microbial community characterization. Much more frequently, the planktonic biogas fermenter microbiome and the impact of OLRs on community structures were studied. In mesophilic AD systems, an increase in OLR supported proliferation of fermentative bacteria of the phyla *Firmicutes*, *Bacteroidota* and *Actinobacteria*. These results are in agreement with the results of the microbial community characterization presented in this study. Apart from fermentative bacteria, the order *Thermoanaerobacteriales*, including several known syntrophic acetate-oxidizing bacteria e.g., *Caldanaerobacter* and *Alkaliphilius*, was shown to increase [32]. The methanogenic archaeal community mainly consisted of members of the genera *Methanosarcina* [33–35]. The *Methanobacteria*, *Methanomicrobiales*, and/or *Methanomassiliicoccaceae* members were also observed in certain AD-processes featuring a high organic load, depending on other prevailing conditions [36].

In thermophilic AD systems, an accumulation of the phyla *Thermotogae*, followed by *Firmicutes* and *Bacteroidota* [34] was shown in reactor systems featuring high OLRs. From the phylum *Thermotogae*, the genus *Defluviitoga* [20, 34, 37] was distinctly abundant. The genera *Ruminiclostridium*, *Herbinix* (both assigned to the *Firmicutes*) and *Caproiciproducens* (novel genus within *Clostridium* Cluster IV) were predominant. As methanogens, species of the genera *Methanosarcina* and *Methanothermobacter* were observed to increase in response to higher OLRs.

Despite the differences in all these biogas community analyses, the response of *Firmicutes* species to changes in OLRs has been shown several times [33, 38]. Members of the phylum *Bacteroidota* were detected in higher abundances in reactors with increased OLRs, much more often only in mesophilic AD [31, 33]. In the HR biofilms analyzed in this study, thermophilic candidates assigned to the order *Bacteroidales,* namely the MAGs 17 (*Prevotella* sp.) and 108 (unknown species), were detected as moderately abundant as well as transcriptionally active in response to the increase in OLRs. Both MAGs are assumed to participate in hydrolysis; however, they were predicted to play a secondary role in the biogas biofilm microbiome. Both MAGs showed significantly increased transcriptional activities when the OLR was raised from 500 to 1500 g. However, in the context of the transcriptional activity of the entire microbial community, the contribution of MAGs 17 and 108 only is of minor importance.

Currently, this is the first study describing the impact of temperature and OLR on the composition of biomass-attached cellulolytic/hydrolytic biofilms and corresponding transcriptional responses of Metagenomically Assembled Genomes (MAGs). The observation, that thermophilic and mesophilic community members behave differently in response to increased OLRs regarding proliferation and transcriptional activity has not been described before and therefore should be further investigated in detail.

## Conclusions

Beside methanogenesis, the ‘bottleneck‘ of biomass AD is the primary hydrolysis of high-molecular carbohydrates such as cellulose, xylan, and other polysaccharides decomposed to volatile fatty acids, short-chained alcohols, and CO_2_ / H_2_ as essential substrates for methanogenesis. High-throughput metagenome analyses enabled the collection of microbiome data to a large scale without the need of time-consuming cultivation [39]. Detailed information on microbiome structure and dynamics in response to process parameters and conditions is accessible by application of corresponding methods. Based on this information, phylogenetic and functional molecular ecological networks (pMEN resp. fMEN) can be developed resulting in the definition of core microbiomes comprising key species for AD [36, 40–42]. Alternatively, a network of metabolic functionality independent from microbial community structure can be established [43].

Based on a combined analysis of metagenome and metatranscriptome datasets, species featuring outstanding performance under increased OLRs were identified. This offers opportunities for biotechnological application of corresponding strains in heavy-duty biogas processes. However, corresponding strategies will require the availability of appropriate isolates. Therefore, high-throughput culturomics approaches as recently developed for human intestinal microbiome analyses [44] should be established also for engineered AD systems yielding isolates featuring the potential to serve as inoculation and / or amendment strains. In addition, isolate-derived genome datasets will support the bioinformatical analysis of metabiome-derived metaomics datasets.

The applied approach is promising for identification of metabolically active AD community members possessing specific, advantageous properties under stress and / or disturbed process conditions without the need for prior cultivation. Insights into the metabolic potential and activity of resilient, robust, and competitive AD species provides the basis for a rational design of their management to counteract process disturbances and to increase methane production rates in challenging fermentation processes utilizing renewable primary products.

## Methods

### Reactor set-up and sampling

Two-stage two-phase biogas reactor systems consisting each of one batch downflow hydrolysis reactor (HR, vol. 10 L), one process fluid storage tank (vol. 10 L), and one downstream upflow AF reactor (vol. 10 L), were operated at mesophilic (M, 37 °C) and thermophilic (T, 55 °C) temperatures and over a period of > 750 d (Fig. 1, Fig. 2). For each reactor system and for each process temperature, two replicates were conducted in parallel, denominated further as biological replicates. Further process details were as previously published by [3]. Start-up of all fermenters were performed using liquid fermenter material from a biogas plant converting cattle manure in co-digestion with grass and maize silage and other biomass at varying concentrations and at mesophilic temperatures.

Silage of perennial ryegrass (*Lolium perenne* L.) was digested as sole substrate in batches of varying amounts with retention times of 28 d (storage of bale silage at − 20 °C, cutting length 3 cm), volatile substances (VS) 32% of fresh mass (FM), total Kjeldahl nitrogen 7.6 g kg_FM_^− 1^, NH_4_^+^-N 0.7 g kg_FM_^− 1^, acetic acid 2.6 g kg_FM_^− 1^, propionic acid < 0.04 g kg_FM_^− 1^, lactic acid 2.6 g kg_FM_^− 1^, ethanol 2.2 g kg_FM_^− 1^, C/N ratio 19.3, chemical oxygen demand (COD) 357.7 g kg_FM_^− 1^, analysis of chemical properties according to [6]. The total Kjeldahl nitrogen is an approximation of a protein content in the sample. The average pH had a value between 7 (at the beginning of every experiment) and 8 (at the end of every experiment after 28 days). No spoilage was observed in the silage. Biogas yields were calculated as liters normalized to 0 °C and 1013 hPa (L_N_) per kilogram volatile substances (kg_VS_). For chemical analysis, samples were taken from the effluents of HR and AF.

For sequencing of 16S rRNA gene amplicon libraries, microbial metagenomes, and microbial metatranscriptomes, samples were taken from the silage digestate in the HR digested for 2 d. At this time point, high AD rates were detected as indicated by the fast increase of volatile fatty acids (VFA), e.g., acetic acid. Sampling was performed at two different organic loading rates (OLRs), i.e.*,* batch-fermentation of 500 g (denominated as “low OLR”, samples M_OLR500_ and T_OLR500_) and 1500 g silage (denominated as “increased OLR”, samples M_OLR1500_ and T_OLR1500_) (Fig. 2). Excess air was removed and the bottle with approximately 200 mg of silage digestate was tightly closed with a screw cap. The biofilm on plant material surfaces originating from the digestate sample was detached using a sterile scalpel in an anaerobic chamber. Further details were described by [12]. All samples were stored at − 20 °C until further analysis except samples for RNA isolation, which were processed immediately after sampling.

### Extraction of total microbial genomic DNA

Total microbial community DNA was extracted from surface attached biofilms by using the FastDNA™ Spin Kit for Soil (MP Biomedicals, USA) with Lysing Matrix E Tubes according to the manufacturer’s instructions. Mechanical cell disruption was performed using the FastPrep®-24 Instrument (MP Biomedicals, USA) for two times at 6500 rpm (speed 5) for 20 s. Further, two washing steps using SEWS-M were accomplished. Finally, the DNA was eluted in 100 μl DES. Two independent technical replicates for each HR were prepared, and subsequently pooled in equimolar amounts together to collect sufficient DNA material for sequencing purposes (Additional file 7). Quality and quantity of extracted DNA were evaluated by gel electrophoresis and photometric analysis (NanoPhotometer, Implen). All DNA samples were stored at − 20 °C until further processing.

### Terminal restriction fragment length polymorphism (TRFLP) fingerprinting

The microbial community dynamics during the operation of the biogas reactor systems were monitored by DNA-based TRFLP analysis targeting the bacterial 16S rRNA gene according to the protocol previously published by [12] with the modifications as published by [45]. TRFLP profiles were determined in triplicates for microbial DNA samples purified from the HR effluent after 28 d batch fermentation. TRFLP fingerprint processing and subsequent analysis were performed according to [46] using BioNumerics 7.1 software (Applied Maths, Belgium). Similarities of fingerprint profiles were calculated using Pearson correlation with 0.5% optimization, cluster analysis was performed applying the unweighted pair group method with arithmetic mean (UPGMA) algorithm.

### Next-generation-sequencing (NGS) of 16S rRNA gene amplicon libraries

The microbial community structures were taxonomically characterized by high-throughput next generation sequencing (NGS) of 16S rRNA gene amplicon libraries as described previously [47]. The libraries were constructed using the primers 515F (5′ - CTACGGGNGGCWGCAG - 3′) and 806R (5′ - GACTACHVGGGTATCTAATCC - 3′) amplifying the V3 and V4 regions of the bacterial 16S rRNA gene [48]. Two biological replicates per sample were analyzed (Additional file 7). Obtained sequence reads were used for iterative read pair merging applying the FLASH software [49]. Subsequently, the QIIME NGS analysis pipeline was applied for amplicon data processing as described previously [29]. Obtained OTUs were clustered at the 97% sequence identity level applying the QIIME NGS analysis pipeline.

### Microbial metagenome library preparation, NGS, and assembly of genomes from metagenome datasets

For library preparation, microbial DNA samples were purified using the Genomic DNA Clean & Concentrator Kit (Zymo Research, USA). For each sample, DNA from two subsamples (i.e., technical replicates) were extracted. For sequencing, 1 μg of total DNA was sheared to approximately 430 bp fragments using a focused-ultrasonicator (Covaris M220, Woburn, MA, USA). Finally, the Illumina TruSeq® DNA PCR-free sample preparation kit (Illumina, Eindhoven, Netherlands) was used to construct the sequencing libraries, which were sequenced on the Illumina HiSeq 1500 sequencer using the Illumina HiSeq Rapid SBS Kit v2 (Illumina, Eindhoven, Netherlands), following a 2 × 250 indexed high output run protocol.

Furthermore, Megahit tool (v1.0.2) [50] (command line settings: --presets meta --min-contig-len 1000) was used for assembly of the pooled sequencing data of all samples applying a k-mer sizes of 21, 41, 61, 81 and 99 (iterative assembly). Paired-end metagenome reads from individual datasets were mapped versus all assembled metagenome contigs with Bowtie 2 (v2.2.4) [51] in end-to-end mode applying the option ‘sensitive’. To convert SAM to BAM, sort the alignment file and calculate read mapping statistics SAMtools (v1.0) [52] was used. Furthermore, to predict genes on assembled contigs larger than 1 kb, the gene prediction tool Prodigal v.2.6.0 [53] was applied. Predicted protein sequences were compared to NCBI’s database using the BLASTP mode of DIAMOND [54]. The resulting output file was loaded into MEGAN5 [55] for taxonomic classification of each gene sequence. In the following binning step, the abundance profile and the tetranucleotide frequencies were used to bin contigs into metagenome-assembled genomes (MAGs) with MetaBAT (v0.21.3) [56]. Mapping of the reads was always performed on all contigs, including the contigs of the MAGs and the contigs which were not binned. Subsequently, completeness, contamination, and strain heterogeneity of the MAGs were estimated with CheckM (v1.0.4) [22], using sets of clade-specific single-copy marker genes.

### Microbial metatranscriptome library preparation and NGS

The total microbial RNA from two sub-samples (from the same silage biofilm as for the DNA extraction) was extracted applying the RNeasy Mini Kit (Qiagen, Hilden, Germany) according to the manufacturer’s guidelines. In total, two technical replicates were processed for each of two parallel hydrolytic reactor communities yielding eight samples for the mesophilic system and eight samples for the thermophilic system (Additional file 7).

Subsequently, the RNA was purified using the Ambion® Turbo DNA-free Kit (ThermoFisher, Germany). Ribosomal RNA was depleted using the Ribo-Zero™ rRNA Removal Kit for Bacteria (Illumina, Madison, USA) according to the manufacturer’s instructions. The remaining mRNA transcripts were fragmented to approximately 550 bp fragments using a focused-ultrasonicator (Covaris M220, Woburn, USA). cDNA libraries for Illumina sequencing were constructed using the TruSeq RNA Library Prep Kit v2 (Illumina, Eindhoven, Netherlands). The resulting cDNA libraries were sequenced on the Illumina HiSeq 1500 machine using the Illumina HiSeq Rapid SBS Kit v2 (Illumina, Eindhoven, Netherlands) to generate 2 × 100 bp paired-end reads.

### Metagenome and metatranscriptome sequence analysis

Paired end metagenome and metatranscriptome reads were mapped versus all assembled metagenome contigs with Bowtie2 [51] in end-to-end mode with option sensitive. After read mapping SAMtools [52] was used to filter the resulting BAM files for uniquely mapped reads. Reads were classified as uniquely mapped reads with a unique genomic location if and only if they could not be aligned to another location with a higher or same mapping quality. Metagenome as well as metatranscriptome reads that could be aligned to assembled MAG’s were quantified with the HTSeq-count program [57] to get an estimate of MAG abundance and overall MAG expression, respectively. To account for different MAG abundance, raw metatranscriptome read counts for each MAG plus one (pseudocount) were divided by the MAG’s raw metagenome read count plus one and rounded to integer values. The resulting counts for each MAG served as input for DESeq2 [58] for pairwise detection and quantification of differentially abundant and transcriptionally active MAGs, respectively. For DESeq2 parametrization, a beta prior and disabled Cook distance cutoff filtering was used. All other parameters remained unchanged. Fold change estimates, *p*-values, and regularized log-transformed (rlog) counts OF (1) metagenome read counts and (2) metatranscriptome read counts normalized for different MAG abundances as described above were emplyed to assess proper sample resp. replicate clustering with (PCA, multidimensional scaling (MDS)), and hierarchical clustering. In addition, volcano plots were used to identify significantly differentially expressed MAGs. Similar comparative analyses were performed assuming equal MAG abundances.

### Phylogenetic and functional analysis of the metagenome-assembled genomes

The Genome Taxonomy Database toolkit [59] was used to assign objective taxonomic classifications to bacterial and archaeal genomes. Each MAG was defined as single operational taxonomic unit (OTU). For MAGs with completeness values of more than 50% and a contamination rate less than 10% (Additional file 4), analysis of the genetic potential was performed using the EMGB [60] annotation system including KEGG pathway mapping and DIAMOND tool [54]. MAGs that meet the criteria mentioned above were subsequently analyzed regarding their transcriptional activities using the EMGB annotation system again.

To predict genes encoding carbohydrate-active enzymes, the carbohydrate-active enzyme database (CAZy) annotation web server dbCAN v7 [24] was used. The genes encoding enzymes acting direct on cellulose were identified by the presence of coding regions for type one or two dockerin or cohesin modules, among others.

## Supplementary information

**Additional file 1.** Statistics of 16S rRNA gene sequence analysis.

**Additional file 2.** Relative abundances of most abundant 16S rRNA gene sequences.

**Additional file 3.** Statistics of the obtained and processed metagenome and metatranscriptome sequences.

**Additional file 4.** Taxonomic affiliations of metagenome-assembled genomes (MAGs) of this study originating from HR biofilms.

**Additional file 5.** Analysis of the key enzymes of AD pathways in metagenome-assembled genomes (MAGs). The genetic determinants were categorized according to the four stages of the AD process, namely hydrolysis, acidogenesis, acetogenesis and methanogenesis as described previously [23].

**Additional file 6.** Hierarchical clustering of abundance values for 78 selected metagenome-assembled genomes (MAGs) detected in HR biofilms at mesophilic and thermophilic process temperature at organic loading rate (OLR) of 500 g resp. 1500 g ryegrass silage as deduced from transcriptome data.

**Additional file 7.** Experimental set up and sampling scheme.

## Data Availability

Sequence datasets were deposited in the European Nucleotide Archive (ENA) under the Bioproject accession numbers PRJEB27769 (metagenome datasets), E-MTAB-7533 (metatranscriptome datasets), PRJEB30260 (16S rRNA gene amplicon sequences).

## Abbreviations

AD: Anaerobic digestion
AF: Anaerobic filter
ANI: Average nucleotide sequence identity
CAZymes: Carbohydrate-active enzymes
CO_2_: Carbon dioxide
COD: Chemical oxygen demand
d: day
FM: Fresh mass
GH: Glycosyl hydrolase
HR: Hydrolysis reactor
HT: High-throughput
kg: kilogram
kg_VS_: kilograms volatile substances
L: Liter
L_N_: Liter normalized
M: Mesophilic
MAG: Metagenome assembled genomes
MG: Microbial metagenome dataset
MT: Microbial metatranscriptome dataset
OLR: Organic loading rates
OTU: Operational taxonomic unit
SAOB: Syntrophic acetate-oxidizing bacteria
T: Thermophilic
TPM: Transcripts per million
TRFLP: Terminal restriction fragment length polymorophism
VFA: Volatile fatty acids
VS: Volatile substances
