# Supplementary figures and images for: Impact of process temperature and organic loading rate on cellulolytic / hydrolytic biofilm microbiomes during biomethanation of ryegrass silage revealed by genome-centered metagenomics and metatranscriptomics
Source: Environ Microbiome. 2020 Mar 2;15:7. doi: 10.1186/s40793-020-00354-x (PMC8067321; doi:10.1186/s40793-020-00354-x)

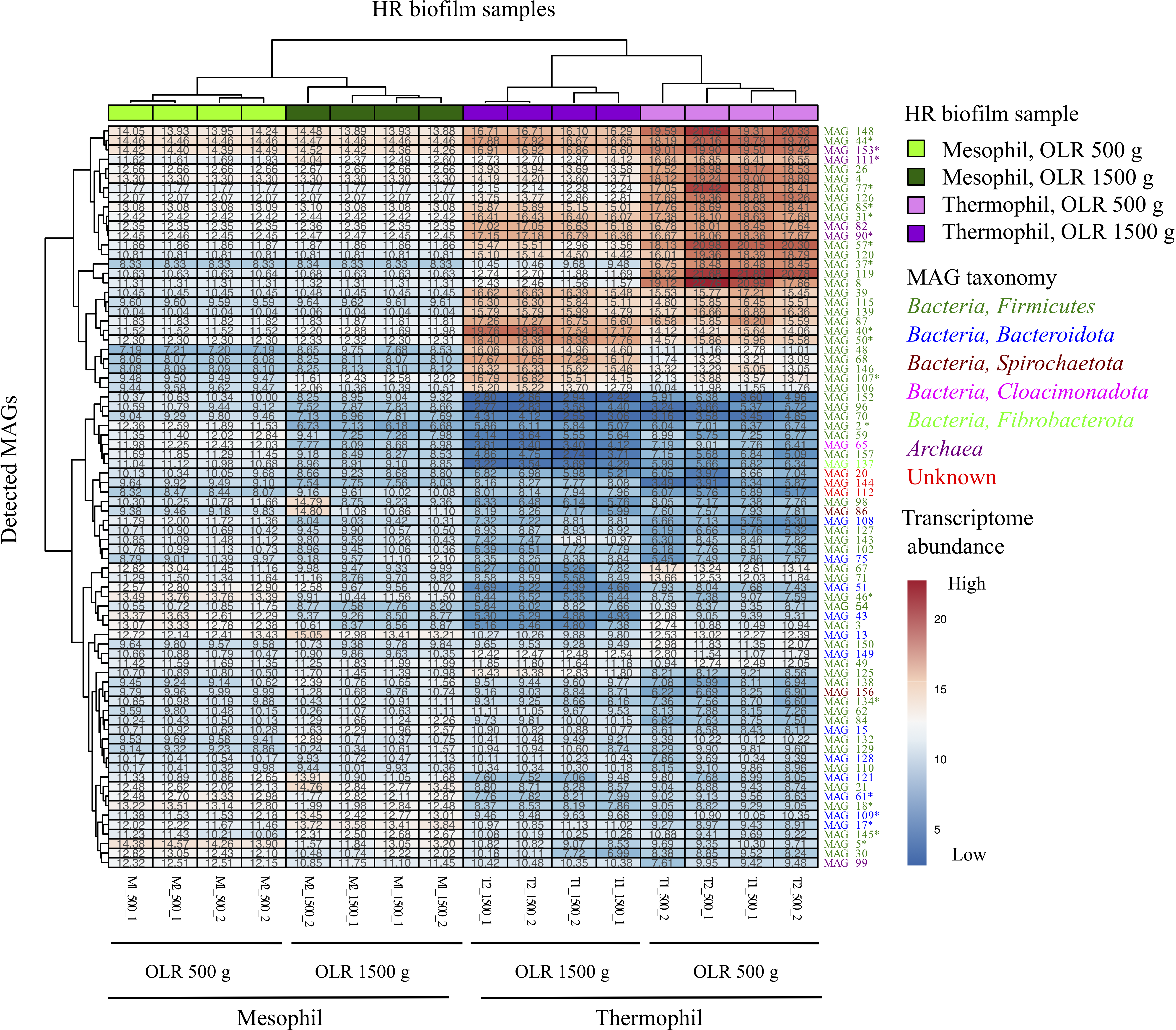

Supplement: Supplementary file 6 — Additional file 6. Hierarchical clustering of abundance values for 78 selected metagenome-assembled genomes (MAGs) detected in HR biofilms at mesophilic and thermophilic process temperature at organic loading rate (OLR) of 500 g resp. 1500 g ryegrass silage as deduced from transcriptome data. [file 40793_2020_354_MOESM6_ESM.tiff]

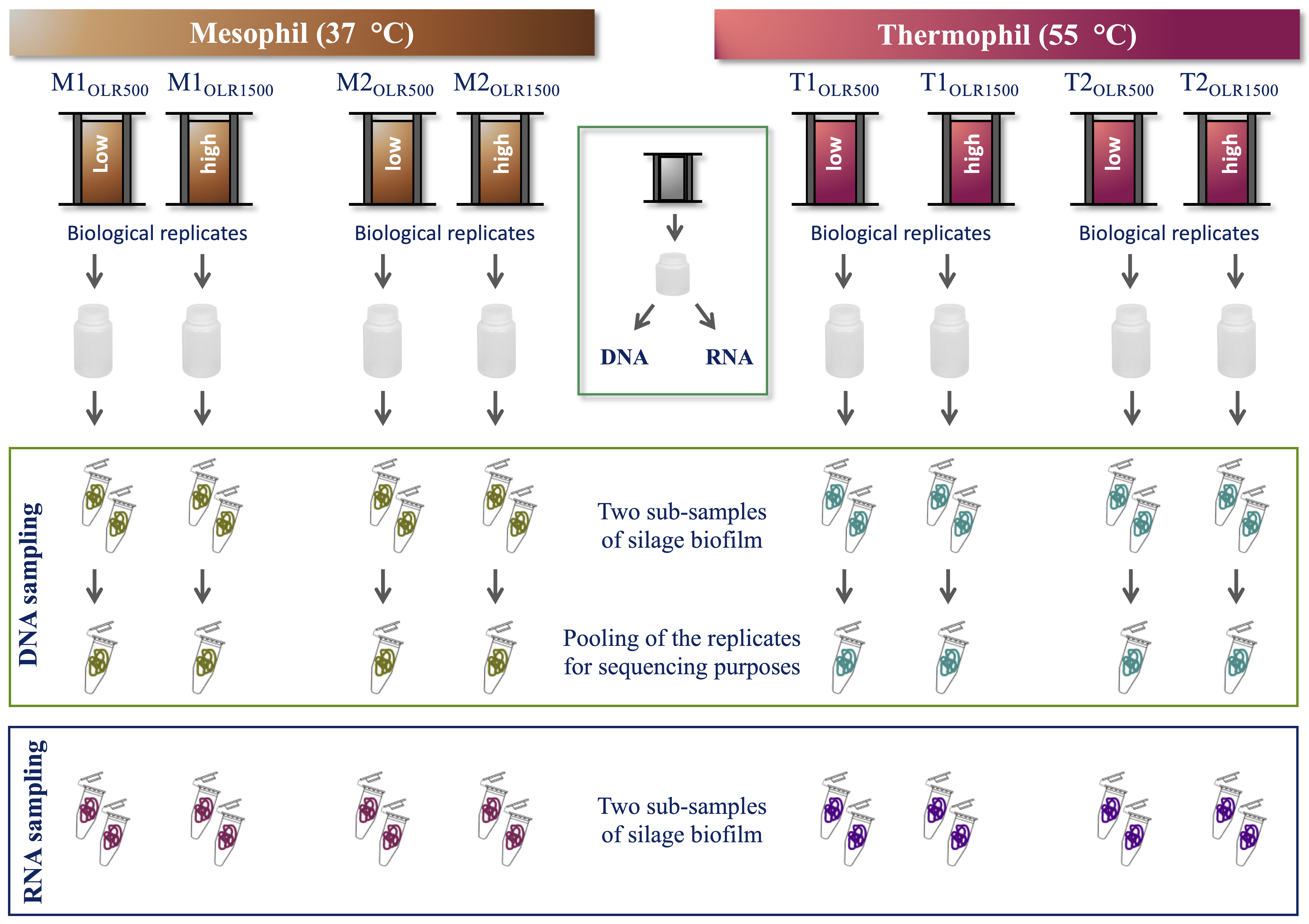

Supplement: Supplementary file 7 — Additional file 7. Experimental set up and sampling scheme. [file 40793_2020_354_MOESM7_ESM.tiff]
